# Safety and efficacy assessment of an mRNA rabies vaccine in dogs, rodents, and cynomolgus macaques

**DOI:** 10.1038/s41541-024-00925-w

**Published:** 2024-07-20

**Authors:** Jianglong Li, Pengcheng Yu, Qi Liu, Long Xu, Yan Chen, Yan Li, Fan Zhang, Wuyang Zhu, Yucai Peng

**Affiliations:** 1 Liverna Therapeutics Inc., Zhuhai, 519000 China; 2 National Institute for Viral Disease Control and Prevention, China CDC, Key Laboratory of Biosafety, National Health Commission, Beijing, 102206 China; 3 AIM Vaccine Co. Ltd., Beijing, 100076 China

**Keywords:** RNA vaccines, Viral infection

## Abstract

Rabies is a lethal disease caused by the rabies virus (RABV), which causes acute neurological infections in mammals, including human beings. We previously reported that an mRNA vaccine (LVRNA001) encoding the rabies virus’s glycoprotein induced strong protective immune responses to rabies in mice and dogs. Here, we further evaluate the safety of LVRNA001. First, we performed a confirmative efficacy study in dogs, which showed that LVRNA001 fully protected the animals from the virus, both pre- and post-infection. Moreover, using pre- and post-exposure prophylaxis murine models, we showed that LVRNA001, built from the CTN-1 strain, was able to protect against various representative RABV strains from the China I–VII clades. To evaluate the safety of the vaccine, chronic and reproductive toxicity studies were performed with cynomolgus macaques and rats, respectively. In a repeated-dose chronic toxicity study, vaccinated monkeys displayed no significant alterations in body weight, temperature, or hematological and biochemical markers. Lymphocyte subset measurement and histopathological examination showed that no toxicity was associated with the vaccine. The immunogenicity study in cynomolgus macaques demonstrated that LVRNA001 promoted the generation of neutralizing antibodies and Th1-biased immune response. Evaluation of reproductive toxicity in rats revealed that administration of LVRNA001 had no significant effects on fertility, maternal performance, reproductive processes, and postnatal outcomes. In conclusion, LVRNA001 can provide efficient protection against rabies virus infection in dogs and mice, and toxicity studies showed no significant vaccine-related adverse effects, suggesting that LVRNA001 is a promising and safe vaccine candidate for rabies prophylaxis and therapy.

## Introduction

Rabies is a widespread, fatal viral zoonosis that infects wild animals, livestock, and humans. It is caused by the rabies virus (RABV)^1^. Rabies is responsible for ~60,000 deaths worldwide each year, the majority of which occur in Africa and Asia. Around 40% of deaths are in children below 14 years of age^2^. RABV has a classic bullet-shaped structure with a 12 kb non-segmented ssRNA genome that codes nucleoprotein (N), phosphoprotein (P), matrix protein (M), glycoprotein (G), and RNA-dependent RNA polymerase (RdRp; also termed large protein, L)^3^. The G protein forms spikes on the viral surface and is the primary target of the host humoral^4^ and cell-mediated immune responses^5,6^. G protein is also used as a target for developing vaccines as it is the only exposed protein on the viral surface, and neutralizing antibodies against G are the correlate of protection against rabies^7^.

Rabies is a vaccine-preventable disease, and prevention by vaccination is the only successful intervention strategy against rabies in areas where dogs are the main source of human infection^8^. Thus, the rabies vaccine is highly recommended for both humans and animals at high risk of exposure and post-exposure. In recent years, numerous studies have concentrated on developing novel, safe, and effective rabies vaccines. Inactivated vaccines, live attenuated vaccines, and recombinant subunit vaccines are widely utilized in the veterinary field, but there are drawbacks to each. Inactivated vaccines require multiple doses to elicit ideal immunity protection, which is a large economic burden in developing countries^9,10^. With live attenuated vaccines, there is the potential risk of strains reverting to a virulent phenotype^11,12^. Recombinant virus vaccines, including the vaccinia virus-based rabies glycoprotein recombinant virus vaccine (V-RG) and the recombinant modified vaccinia virus Ankara (MVA) expressing a copy of the rabies virus glycoprotein gene, have been reported to present limited protection^13^. Efforts focusing on developing safe, cost-effective, and sustained immunogenic rabies vaccines are necessary.

Messenger RNA (mRNA) vaccine technology is a rapid vaccine platform that has shown promise against cancer and infectious diseases^14,15^. Cell-free in vitro transcription of mRNA is scalable, as made evident during the SARS-CoV-2 pandemic^16^. Through a lipid-based delivery system, mRNA vaccine-encoding immunogens are delivered to target cells and elicit vaccine-specific responses^14^. mRNA vaccines are quick and cost-efficient to produce, and have flexibility in immunogen design^17–19^. In addition, mRNA vaccines can provide a safe and controllable pattern of gene expression without integration into the host genome^19^. The safety and immunogenicity of mRNA vaccines have been previously reported in humans and animals, including rabies mRNA vaccines^20–22^.

Non-replicating mRNA vaccines expressing RABV-G were developed in the past few years, and have shown good protective effects and tolerability in animal and clinical studies^21,23,24^. For example, the RABV-G-expressing rabies mRNA vaccine RV021 was confirmed to induce a strong protective immune response in mice^25^. An mRNA-based vaccine encoding rabies virus glycoprotein has been demonstrated to achieve protective virus-neutralizing titers in preclinical studies^22,26^ and phase 1 clinical trials^21^. Additionally, a single dose of nucleoside-modified RABV-G mRNA vaccine (RABV-G mRNA-LNP or LPP-mRNA-G) has been shown to confer effective protection and elicit potent humoral immunity^27,28^. Unfortunately, no commercial rabies mRNA vaccine is available so far. In all reported RABV mRNA vaccination studies, outstanding protective immune responses were induced in mice and dogs. However, a more clinically relevant animal model, such as non-human primates, has not been used to evaluate the safety and immunogenicity of mRNA vaccines. We previously reported that the rabies mRNA vaccine candidate (LVRNA001), which encodes the G antigen, induced strong protective immune responses in mice and dogs^23^. In the current study, we present further confirmative efficacy results and systematic safety evaluation of the product with cynomolgus macaques and rats.

## Results

### LVRNA001 vaccination provided complete protection in pre- and post-exposure treatment of RABV-infected dogs

**Fig. 1:**
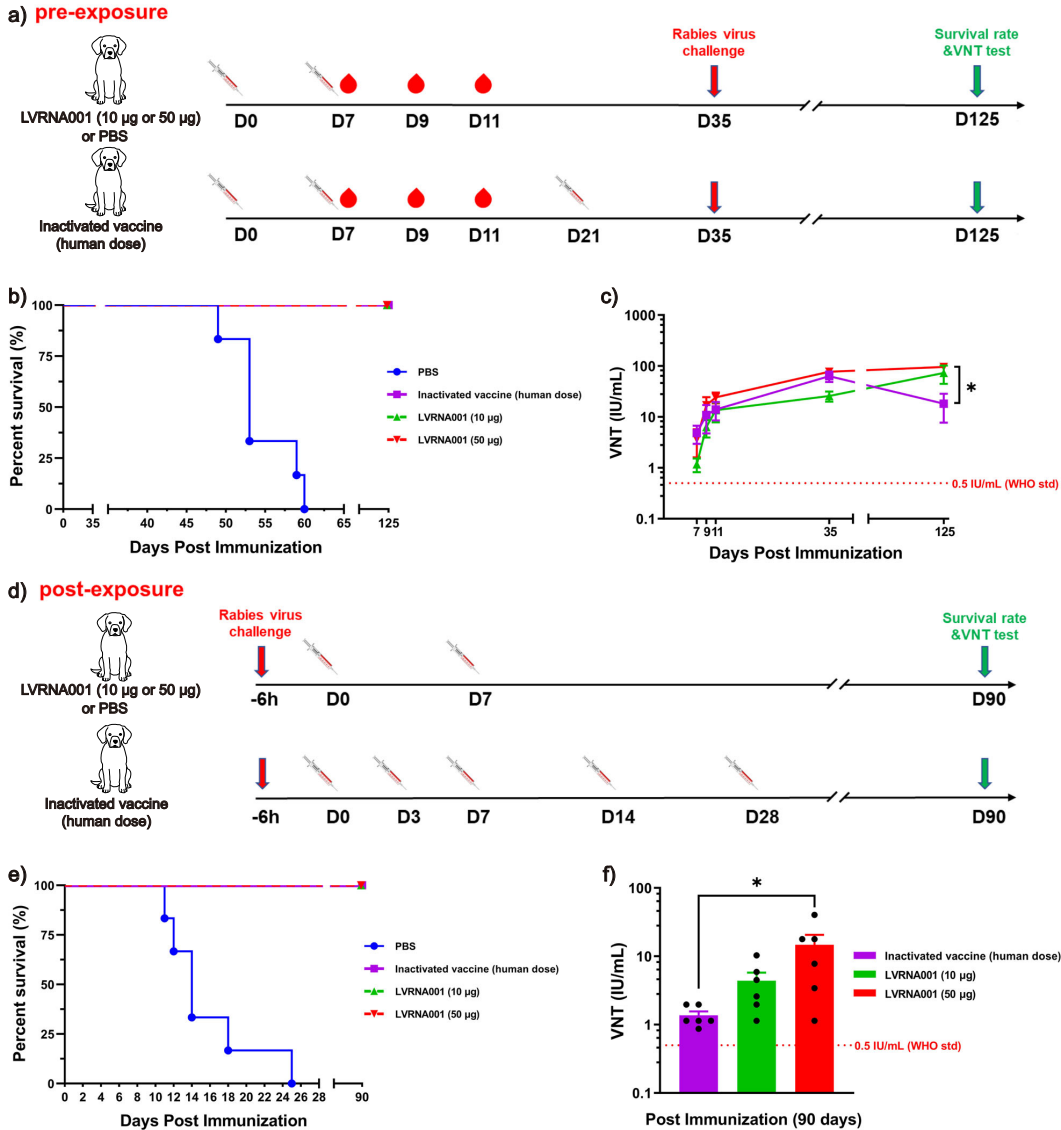
LVRNA001 provided complete protection to RABV-infected dogs in pre- and post-exposure treatment. **a** Schematic diagram depicting the pre-exposure treatment schedule for dogs. Dogs were vaccinated i.m. with 10 μg or 50 μg of LVRNA001 (0d/7d), inactivated vaccine (0d/7d/21d), or PBS. All dogs were challenged with 50-fold LD_50_ of street virus BD06 strain on D35. **b** Survival curves until 125 days after vaccination. *n* = 6. **c** Neutralizing antibody levels in dogs were detected using FAVN on D7 (day 7), D9, D11, D35, and D125 post-vaccination. *n* = 6. Error bars represent standard deviation. Statistical analysis was performed using an ordinary one-way ANOVA. *, *p* < 0.05. **d** Schematic diagram depicting post-exposure treatment schedule for dogs. Dogs were challenged with 50-fold LD_50_ of the virulent RABV-BD06 strain. Six hours later, high-dose (50 μg) and low-dose (10 μg) groups were vaccinated i.m. with LVRNA001 (0d/7d), while the positive control group received five doses of inactivated vaccine (0d/3d/7d/14d/28d), or PBS as a negative control. **e** Survival curves are shown out to 90 days post vaccination with a two-day interval. *n* = 6. **f** Neutralizing antibody levels were analyzed at D90. *n* = 6. Error bars represent standard deviation. Statistical analysis was performed using an ordinary one-way ANOVA. *p < 0.05.

In 2010, the World Health Organization (WHO) stated that pre-exposure prophylaxis guideline was one-site vaccination over 3 days, day 0, 7, 21/28 (D0-7-21/28 immunization schedule), whereas post-exposure prophylaxis guidelines included three intramuscular regimens: the five-dose Essen regimen (1-1-1-1-1, 5 days, D0-3-7-14-28 immunization schedule), the four-dose Zagreb Regimen (2-1-1, two-site vaccination on day 0 and one-site vaccination on days 7 and 21), and the four-dose shortened Essen regimen (4 days, D0-3-7-14 immunization schedule). The current 2018 WHO guidelines recommend a shorter intramuscular pre-exposure prophylaxis regimen (2 days, D0-7 immunization schedule) compared to previous guidelines (3 days, D0-7-21/28). Current 2018 WHO post-exposure prophylaxis guidelines retain the shortened Essen regimen (4 days, one-site vaccination on days 0, 3, 7, wherein the fourth dose can be given at any time between days 14 and 28) and the three-visit Zagreb intramuscular regimen (2-1-1)^29^. However, the Centers for Disease Control and Prevention (CDC) still used the earlier WHO recommendations (2010 WHO guidelines) for inactivated vaccine immunization. In the current study, to highlight the advantages of mRNA vaccines, we first measured the in vivo protective efficacy of LVRNA001 in dogs. Prior to infection with a lethal dose of RABV, dogs were immunized twice (0d/7d, D0-7 immunization schedule) with 10 μg or 50 μg of LVRNA001. Control dogs were vaccinated three times (D0-7-21 immunization schedule) with one human dose of either inactivated vaccine or PBS. All dogs were challenged with 50-fold LD_50_ of street virus BD06 strain on D35 (Fig. 1a). The survival rate of high- and low-dose LVRNA001 and inactivated vaccine groups was 100%, while the survival rate in the PBS group was 0% (Fig. 1b). All vaccinated groups developed high neutralizing antibody levels on D7, D9, D11, D35, and D125, all above the WHO suggested protective threshold (0.5 IU/mL). However, LVRNA001 vaccination groups maintained higher neutralizing antibody titers than the inactivated vaccine group on D125 (Fig. 1c), suggesting that LVRNA001 vaccination generates a stronger response in comparison with the inactivated vaccine.

Next, the vaccine efficacy of post-exposure was assessed. Dogs were divided into four groups and infected via intramuscular (i.m.) injection with 50-fold LD_50_ of virulent RABV-BD06 strain. Six hours after viral infection, dogs were vaccinated i.m. with LVRNA001 (10 or 50 μg, D0-7), or one human dose of inactivated vaccine (D0-3-7-14-28) as a positive control, or PBS as a negative control (Fig. 1d). Survival rates of 90 days post-vaccination revealed that dogs vaccinated twice with 10 or 50 μg of LVRNA001, and five times with inactivated vaccine, exhibited 100% survival, whereas all dogs receiving PBS died (Fig. 1e). Three months post-vaccination, relatively higher neutralizing antibody levels were detected in high-dose and low-dose groups compared with those measured in the inactivated vaccine group (Fig. 1f).

### Vaccination schedule exploration of LVRNA001 in mice

**Fig. 2:**
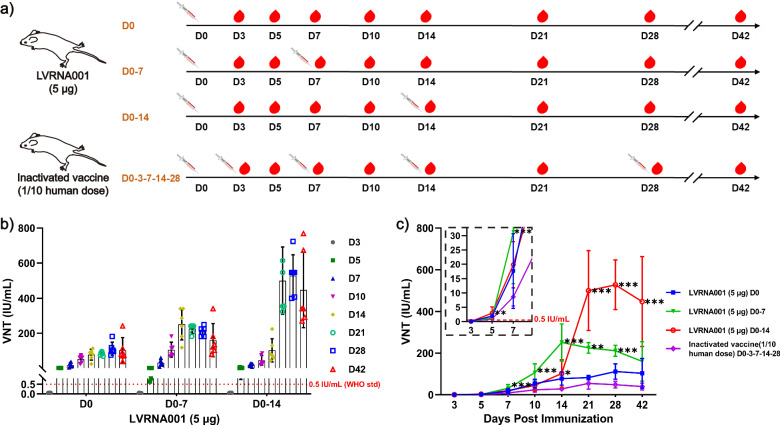
Vaccination schedule exploration of LVRNA001 in mice. **a** Schematic diagram depicting the experimental procedure. Mice were vaccinated i.m. with LVRNA001 (5 μg, 0d or 0d/7d, or 0d/14d), or inactivated vaccine (1/10 human use, 0d/3d/7d/14d/28d). **b**, **c** Sera were collected on D3, D5, D7, D10, D14, D21, D28, and D42 after the first vaccination, and neutralizing antibody levels in the LVRNA001 high-dose group (5 μg) were detected using RFFIT test. *n* = 6. Error bars represent standard deviation. Statistical analysis was performed using an ordinary one-way ANOVA. *p < 0.05. **p < 0.01. ***p < 0.001. “*” vs inactivated vaccine.

To investigate the effect of LVRNA001 after using different vaccination schedules on neutralizing antibody production, mice were vaccinated with LVRNA001 (5 μg, D0 or D0-7 or D0-14), or inactivated vaccine (1/10 human use, D0-3-7-14-28). We collected sera at days 3, 5, 7, 10, 14, 21, 28, and 42 post-vaccination and measured neutralizing antibody titers (Fig. 2a). More neutralizing antibodies were induced by 5 μg of LVRNA001 in the D0-7 (from D7) and D0-14 (from D14) immunization schedules than in the D0 immunization schedule, in the D0-14 (from D21) immunization schedule than in the D0-7 immunization schedule (Fig. 2b), suggesting that booster dosage is important for LVRNA001, and the D0-14 immunization schedule is superior to D0-7 immunization schedule. In addition, we found that administering two doses of LVRNA001 induced significantly higher levels of neutralizing antibodies compared to the inactivated vaccine, even though the latter was administered five times by D42 (Fig. 2c). Interestingly, a single vaccination of LVRNA001 (D0) produced more virus-neutralizing antibodies than two doses (D0-3) of an inactivated vaccine on D5. Neutralizing antibody levels induced by LVRNA001 also exceeded 0.5 IU/mL at an earlier time point than those induced by the inactivated vaccine (Fig. 2c), suggesting that LVRNA001 could stimulate mice to generate higher neutralizing antibody levels at a faster rate than inactivated vaccines.

### LVRNA001 provided protection against RABV in pre- and post-exposure mouse models

**Fig. 3:**
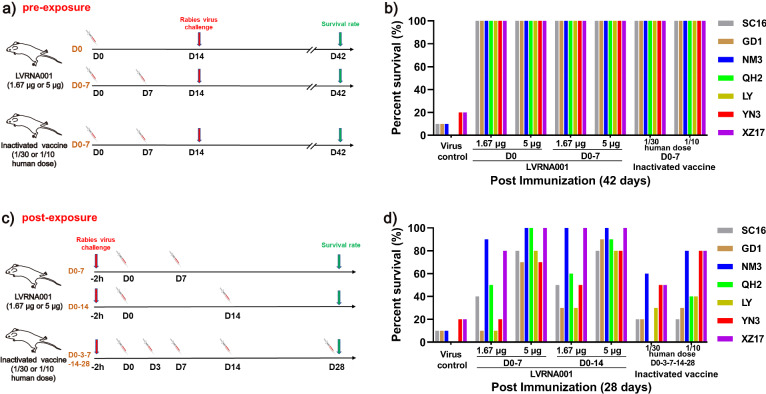
LVRNA001 provided protection against various RABV strains in pre- and post-exposure mouse models. **a** Schematic diagram depicting the experimental procedure for pre-exposure treatment schedule for mice. Mice were vaccinated i.m. with LVRNA001 (1.67 μg or 5 μg, 0d or 0d/7d), or inactivated vaccine (1/10 or 1/30 human use, 0d/7d). Then, they were challenged i.m. with 50-fold LD_50_ of RABVs (SC16, GD1, NM3, QH2, LY, YN3, and XZ17, belonging to China I–VII clades, respectively) on D14 after vaccination. **b** Animal survival was calculated on D42. **c** Schematic diagram depicting the experimental procedure of post-exposure treatment schedule for mice. After challenge i.m. by 50-fold LD_50_ of different RABV clades (SC16, GD1, NM3, QH2, LY, YN3, and XZ17) for 2 h, mice were vaccinated i.m. with LVRNA001 (1.67 μg, 0d/7d or 0d/14d), LVRNA001 (5 μg, 0d/7d or 0d/14d) or inactivated vaccine (1/10 or 1/30 human use, 0d/3d/7d/14d/28d). **d** Animals were monitored and survival rate was evaluated on D28. *n* = 10.

To confirm the better protective effects of LVRNA001, we first evaluated the efficacy of LVRNA001 in a pre-exposure mouse model. Mice were vaccinated i.m. with either different doses of LVRNA001 (1.67 or 5 μg) using D0 or D0-7 immunization schedules, or an inactivated vaccine (1/10 or 1/30 human use, D0-7 immunization schedule) (Fig. 3a). At Day 14 after the first vaccination, mice were challenged with an i.m. of 50-fold LD_50_ of RABVs (China I–VII clades) and survival rates were measured on D42. The animals were monitored daily, and survival rates were calculated (Supplementary Fig. 1a–g). We found that the survival rates in LVRNA001 and inactivated vaccine groups were 100%, whereas the overall average survival rate without vaccination in virus groups (strains of SC16, GD1, NM3, QH2, LY, YN3, and XZ17) was 10% (7/70) (Fig. 3b).

Next, the efficacy of LVRNA001 was assessed in a post-exposure mouse model. The mice were infected i.m. with 50-fold LD_50_ of RABVs (SC16, GD1, NM3, QH2, LY, YN3, and XZ17), and then either vaccinated twice (D0-7 or D0-14) with LVRNA001 (1.67 μg or 5 μg), or five times (D0-3-7-14-28) with an inactivated vaccine (1/10 or 1/30 human use) (Fig. 3c). The animals were monitored daily and survival rates were calculated (Supplementary Fig. 2a–g). For LVRNA001, the survival rates in the high-dose group (5 μg) using the D0-7 or D0-14 immunization schedule were 85.71 and 88.57%, respectively, whereas the low-dose group (1.67 μg) exhibited 42.86% (D0-7) and 60.00% (D0-14) survival rates. The survival rates of mice vaccinated with five doses of inactivated vaccine (1/10 or 1/30 human use) were 52.86 and 32.86%, respectively (Fig. 3d), indicating that two dosages of LVRNA001 provided better protection to mice than the inactivated vaccine after exposure to diverse RABVs (China I–VII clades). Consistent with previous results, the two-week dosing interval (D0-14) of LVRNA001 was more effective than the 1-week dosing interval (D0-7).

### LVRNA001 had no adverse effects in an acute toxicity study in mice

To evaluate the toxicity of LVRNA001 in mice, a single-dose, acute toxicity study was carried out. As shown in Supplementary Fig. 3a, mice were injected (i.m.) with a low dose (10 µg) or a high dose (50 µg) of LVRNA001, or Saline on D0. The general clinical signs of the mice were observed and body weight was measured for 14 days. The results revealed no significant differences in body weight (Supplementary Fig. 3b) and weight gain rate (Supplementary Fig. 3c) between the LVRNA001 immunized groups and the PBS control group.

### Biochemical assessment of cynomolgus macaques vaccinated with various doses of LVRNA001

**Fig. 4:**
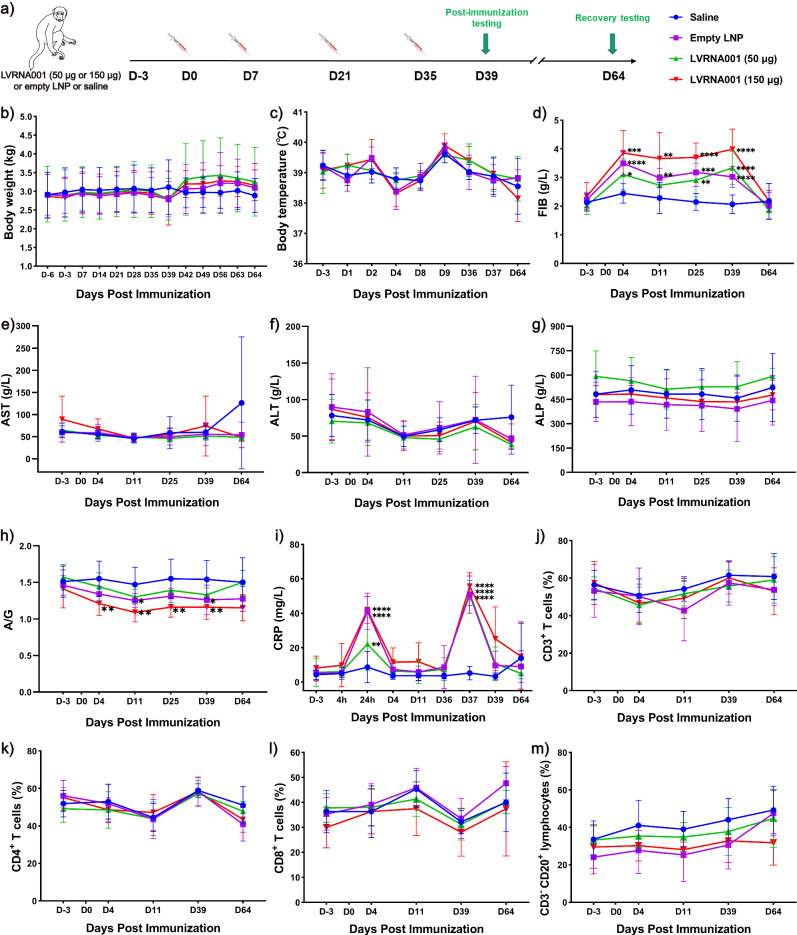
Biochemical assessment and lymphocyte subset measurement of cynomolgus macaques vaccinated with various doses of LVRNA001. **a** Schematic diagram depicting the experimental treatment schedule for cynomolgus macaques. Cynomolgus macaques were injected with LVRNA001 at a low dose of 50 μg, a high dose of 150 μg, and empty LNP or Saline on D0 (0 days), D7, D21, and D35. Data were collected at the indicated time points during a total period of 9 weeks. **b** Body weight was monitored. **c** Temperature was measured. **d** Serum FIB level. **e** Serum AST level. **f** Serum ALT level. **g** Serum ALP level. **h** Serum A/G level. **i** Serum CRP level. Frequencies of CD3^+^ (T-lymphocytes) cells (**j**), CD3^+^CD4^+^ (helper T-lymphocytes) cells (**k**), CD3^+^CD8^+^ (cytotoxic T-lymphocytes) (**l**), and CD3^−^CD20^+^ (B-lymphocytes) cells (**m**) were examined through flow cytometry. FIB fibrinogen, AST aspartate aminotransferase, ALT alanine aminotransferase, ALP alkaline phosphatase, A/G albumin/globulin ratio, CRP C-reactive protein. *n* = 10, except for D64 (*n* = 4). All Error bars represent standard deviation. *P* values were calculated using a two-way ANOVA (**d**, **h**, **i**). *p < 0.05. **p < 0.01. ***p < 0.001. ****p < 0.0001. “*” vs Saline.

To assess the safety of LVRNA001, we explored the effect of LVRNA001 administration on cynomolgus macaques. The animals were injected i.m. with LVRNA001 at a low dose of 50 μg, a high dose of 150 μg, an Empty LNP, or Saline. Each monkey was injected four times over the course of 5 weeks, at days 0, 7, 21, and 35. We collected sera and monitored the monkeys for a total of 9 weeks, until day 64 after the first injection (Fig. 4a). All cynomolgus macaques had similar body weights and temperature (Fig. 4b, c). We registered no changes in biochemical markers of liver function, such as aspartate aminotransferase (AST), alanine aminotransferase (ALT), and alkaline phosphatase (ALP), throughout the whole procedure (Fig. 4e–g). The factors influenced by vaccination were: (1) coagulation-related indicator fibrinogen (FIB), which was elevated in the high and low-dose LVRNA001 groups and empty LNP group after repeated LVRNA001 administration, but restored after a 4-week convalescence period (Fig. 4d), suggesting that FIB increase is related to LNP components of the vaccine and its effects are transient; (2) albumin/globulin ratio (A/G) values, which decreased during the 5-week repeated LVRNA001 administration period but returned to normal level after a 4-week convalescence (Fig. 4h); (3) C-reactive protein (CRP), a non-specific diagnostic marker of inflammation, which increased at 24 h and D37 in the three groups receiving lipid nanoparticles (LNPs) in comparison with saline. These time points are close to the injections and the transient increase reflects most probably the normal inflammation upon injection (Fig. 4i). These results indicated that LVRNA001 is well tolerated by cynomolgus macaques and has no noticeable toxic side effects.

### LVRNA001 did not induce lymphocyte subset variation in cynomolgus macaques

To investigate the effect of LVRNA001 on the immune systems of cynomolgus macaques, we measured lymphocyte subsets on days 3, 4, 11, 39, and 64 after the first LVRNA001 injection. The percentages of T- and B-lymphocyte subpopulations (CD3^+^, CD3^+^CD4^+^, CD3^+^CD8^+^, CD3^−^CD20^+^) were analyzed using flow cytometry. The gating strategy for flow cytometry is shown in Supplementary Fig. 4. The frequencies of T cells (all CD3^+^ T cells), T-helper cells (CD3^+^CD4^+^ T cells), T-cytotoxic cells (CD3^+^CD8^+^ T cells) and CD3^−^CD20^+^ (B-lymphocytes) cells were stable during the 9-week period of observation (Fig. 4j–m), suggesting that LVRNA001 did not significantly affect lymphocyte subsets.

### Histological analysis of tissues of cynomolgus macaques injected with LVRNA001

**Fig. 5:**
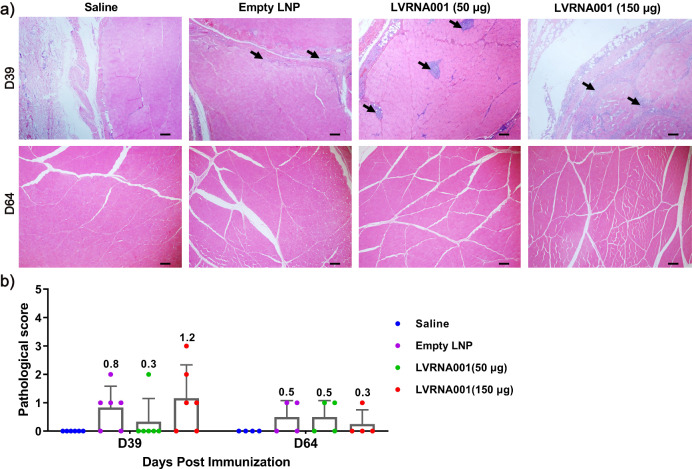
Histological analysis of the site of injection of LVRNA001 in cynomolgus macaques. Cynomolgus macaques were injected four times (0d/7d/21d/35d) with LVRNA001 (50 μg or 150 μg), empty LNP, or Saline. The injection site muscles were collected on D39 and D64. **a** H&E staining was performed for the histopathological examination. The black arrows indicate inflammation. Scar bar, 200 μm. **b** Pathological score was calculated on D39 (*n* = 6) and D64 (*n* = 4). Error bars represent standard deviation. Comparisons among groups were performed using an ordinary one-way ANOVA.

To further explore the effect of LVRNA001 on the organs of cynomolgus macaques, we performed hematoxylin and eosin (H&E) histological analyses of 54 tissues such as brain, heart, liver, spleen, lung, kidney, site of injection muscles, eyeball, optic nerve, and testis, that were collected on days 39 and 64 of the above described experiment. For all tissues except the muscle where the vaccine was injected, the histopathological examination revealed no signs of pathological lesions (data not shown). Mild inflammation was observed at the site of injections in the empty LNP and LVRNA001 groups, while no inflammation was found in the saline-injected group on day 39. Signs of inflammation were no longer present at the site of injection on day 64 (Fig. 5a), meanwhile, pathological scores for the injection site were low for both D39 and D64 (Fig. 5b), suggesting that the inflammation was mild.

### LVRNA001 induced a potent immune response in cynomolgus macaques

**Fig. 6:**
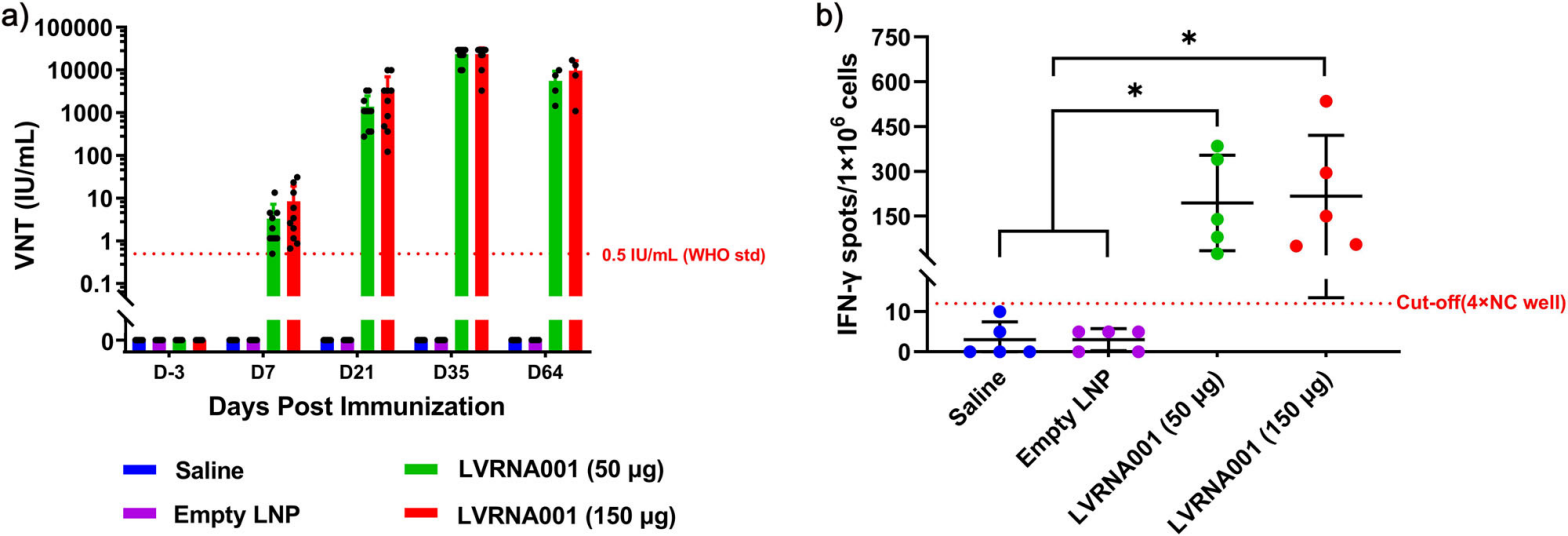
LVRNA001 was highly immunogenic in cynomolgus macaques. Cynomolgus macaques were injected with LVRNA001 at a low dose of 50 μg, a high dose of 150 μg, empty LNP, or Saline on D0, D7, D21, and D35. **a** Viral neutralization titers from serum samples was detected through FAVN on D3, D7, D21, D35, and D64. *n* = 10, except for D64 (*n* = 4). **b** IFN-γ secreting cells were examined using ELISPOT assay on D39. *n* = 5. All error bars represent standard deviation. Statistical analysis was performed using an ordinary one-way ANOVA. *p < 0.05.

Next, we evaluated the immune responses that were elicited by the vaccination of cynomolgus macaques with LVRNA001 (repeated administration during 5 weeks followed by a 4-week convalescence period). Blood samples obtained on D3, D7, D21, D35, and D64 were analyzed for viral neutralization antibody titers by a fluorescent antibody virus neutralization test (FAVN). We found that cynomolgus macaques vaccinated with 50 or 150 μg of LVRNA001 generated strong neutralizing antibody activity, with titers reaching as high as >20000 IU/ml on D35, and remained stable on D64 (Fig. 6a). Antigen-specific T cells induced by LVRNA001 were analyzed by an ELISpot assay on D39. We detected significantly more IFN-γ secreting cells in mRNA immunized cynomolgus macaques compared to Empty LNP or Saline treated animals (Fig. 6b).

### LVRNA001 caused no toxic effects in a reproductive toxicity study in rats

**Fig. 7:**
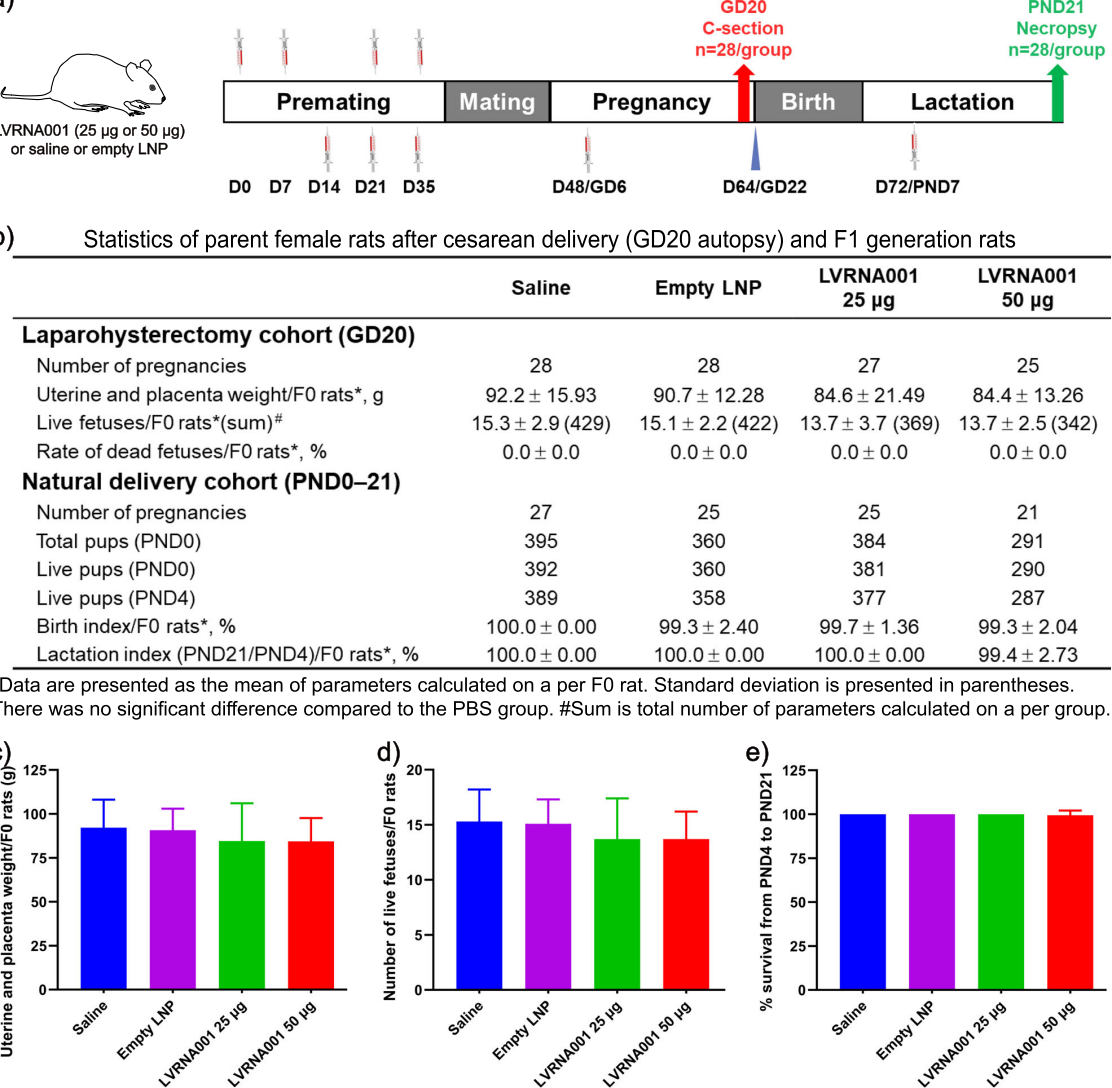
LVRNA001 caused no toxic effects in a reproductive toxicity study in rats. **a** Schematic diagram depicting the reproductive toxicity study schedule for rats. F0 males were immunized i.m. with LVRNA001 (25 or 50 μg), Empty LNP, or Saline on D0, D7, D21, and D35. EFD phase F0 females were immunized i.m. with LVRNA001 (25 or 50 μg), Empty LNP, or Saline on D14 (28 days prior to pairing for mating), D21, D35, and on gestation day 6 (GD6). After mating, littering phase F0 females received LVRNA001 (25 or 50 μg), Empty LNP, or Saline on D14 (28 days prior to pairing for mating), D21, D35, GD6, and postnatal day 7 (PND7). **b** Statistics of parent female rats after cesarean delivery (GD20 autopsy) and F1-generation rats. **c** Uterine and placenta weight. **d** The number of live fetuses. **e** Lactation index (the proportion of pups alive on PND4 who were alive on PND21). Error bars represent standard deviation (**c**, **d**, **e**). Statistical analysis was performed using an ordinary one-way ANOVA.

Next, we conducted a reproductive toxicity study in rats. We tested the effect of LVRNA001 during two phases of the reproductive circle: the embryo-fetal development (EFD) phase and the littering phase. Mating males were treated on D0, D7, D21, and D35 with LVRNA001 (25 or 50 μg). In the EFD phase study, F0 females in each group received LVRNA001 (25 or 50 μg), Empty LNP, or Saline on D14 (28 days prior to pairing for mating), D21, D35, and gestation day (GD) 6, and then experienced laparohysterectomy on GD20 (Fig. 7a). We found that the number of pregnancies, the uterine and placenta weight, the number of live fetuses/F0 rats, and the rate of dead fetuses were similar between groups (Fig. 7b–d). In the littering phase study, which monitored the survival of offsprings, we administered an additional dose of either vaccine or controls on postnatal day (PND) 7. F0 females delivered naturally and were co-housed with offsprings until PND21 (Fig. 7a). Assessments made on PND0 and PND4 showed that the number of pups (relative to pregnancies) that survived was similar among the groups. In summary, LVRNA001 administration did not affect the birth index and lactation index in rats (Fig. 7b, e). These results indicate that LVRNA001 vaccination is safe during pregnancy and lactation periods in rats.

## Discussion

Herein, we confirmed the protection efficacy of various doses of LVRNA001 against lethal doses of RABV in dogs and mice, and evaluated the safety of LVRNA001 in cynomolgus macaques and rats for repeated-dose chronic toxicity and reproductive toxicity studies, respectively. We found that LVRNA001 could elicit powerful immunogenic responses in cynomolgus macaques while maintaining a relatively safe profile.

Currently, the administration of five-dose Essen regimens (1-1-1-1-1) or abbreviated 4-dose Zagreb regimens (2-1-1) of inactivated rabies vaccines have mainly been used for post-exposure rabies vaccination and have exhibited relatively good safety and immune efficacy in preventing human rabies^30^. Previously, we reported that dogs immunized with LVRNA001 (25 μg) for two doses in post-exposure treatment exhibited a 100% survival rate and high neutralizing antibody levels; however, the survival rates of the 5-μg dosage groups was only 83.33%^23^. In the current study, we further confirmed that two doses of LVRNA001 (10 or 50 μg) in post-exposure treatment provided 100% protection, indicating that two dosages of LVRNA001 is enough to provide full protection for dogs.

To explore optimal immunization schedules and cross-strain protection, we demonstrated that D0-7 and D0-14 immunization schedules induced better antibody outcomes than D0 single-dose immunization. On the other hand, a single vaccination with LVRNA001 could induce higher neutralizing antibody titers, and at a faster rate (Day 5) in mice compared with an inactivated vaccine. In the cross-strain challenge study, we confirmed that vaccination with LVRNA001 could provide efficient protection against various viral strain challenges when administered both pre- and post-exposure. Consistent with other results^31^, administration of LVRNA001 provided better protection against different RABV strains (China I–VII clades) than five doses of inactivated vaccine in post-exposure treatment of mice. The survival rate with LVRNA001 vaccination (5 μg, D0-14 immunization schedule) was 88.57%, which was higher than that of the inactivated vaccine (52.86%). Interestingly, although the mRNA and inactivated vaccines had a drastic difference in mice (Fig. 2c), they produced neutralizing antibodies at a similar rate pre-challenge in dogs (Fig. 1c). We noticed similar reports from other research groups. For example, a recent study showed that immunization with LPP-mRNA-G induced significantly higher neutralization antibodies in mice than those from inactivated vaccine, but the neutralization antibody production in LPP-mRNA-G immunized or inactivated vaccine immunized dogs was not significantly different^28^. The detailed mechanism behind this species discrepancy is unclear.

Safety assessment is mandatory for the Investigational New Drug (IND) application. Previous studies reported concerns in clinical studies of rabies vaccine candidates CV7201 and CV7202^21,32^. According to literature, CV7201 requires intradermal or intramuscular administration with specialized devices to induce adequate immune responses^32^; while early stage clinical investigation of CV7202 found unfavorable reactogenicity and immunogenicity profiles with 5 μg dose^21^. Both CV7201 and CV7202 were developed years ago when mRNA technology was still on its path toward maturity. It is not clear if the quality specifications of CV7201 and CV7202 met current regulatory standards. In our acute toxicity study with mice, a single administration of LVRNA001 (10 or 50 µg) did not induce significant toxic signs. A repeated-dose toxicity study with cynomolgus macaques also revealed that both the low and high doses were well tolerated. The vaccine caused no significant change in AST, ALT, and ALP levels in cynomolgus macaques, indicating that repeated administration of LVRNA001 did not change the function and metabolism of hepatocytes. However, repeated administration of LNP or LNP-mRNA caused transient increases of FIB and CRP, but a decrease in the A/G ratio. FIB and CRP as inflammatory markers are often elevated in response to inflammation^33,34^. CRP was reported to be part of the acute phase response, in which many plasma protein synthesis was up-regulated, whereas a smaller number, particularly albumin, was down-regulated^35^. It was also reported that renal inflammatory responses facilitate renal excretion of albumin^36^ and decrease serum albumin levels. We believe that LNP and LNP-mRNA cause inflammation during the 5-week repeated administration, leading to significant changes in markers such as FIB, CRP, and A/G ratio. These changes disappeared after a 4-week convalescence, suggesting a transient inflammatory reaction.

Studies with cynomolgus macaques also showed that LVRNA001 could induce a strong immunogenic response, as evidenced by extremely high neutralizing antibody levels. It is worth noting that 7 days after a single dose of LVRNA001, neutralizing antibody levels were well above the WHO-recommended threshold. Previous studies revealed that T cell immune responses, especially Th1-biased immune response, are important to eliminate RABV viruses from the central nervous system^37,38^. Our study with cynomolgus macaques showed that the T cell response was Th1 polarized with the production of IFN-γ secreting cells. These findings demonstrated that repeated administration of LVRNA001 to cynomolgus macaques causes no systemic toxicity while generating strong specific immune responses against the rabies virus.

To investigate whether there are any vaccine-related adverse effects on maternal processes, we carried out a two-phase reproductive toxicity study, with an EFD phase and a littering phase. Our results showed that the vaccine had no effects on fertility, fetal and neonatal development, or postnatal outcomes in female rats vaccinated with LVRNA001 three times prior to mating, once during gestation and once after delivery. Male rats immunized with LVRNA001 four times prior to mating also showed no adverse effect on male fertility. These results demonstrated that LVRNA001 administration did not affect pregnant rats or their offspring, nor produce reproductive or developmental toxicity.

Taken together, our mRNA-based LVRNA001 can achieve efficient neutralizing antibody levels and provide protection in vivo. The efficacy and safety data presented in this report demonstrated that LVRNA001 is an advantageous vaccine candidate for rabies. It would be important to find out whether clinical studies in humans will reach the same conclusions as preclinical studies.

## Materials and methods

### Cells and viruses

BHK-21 cells and BSR cells were cultured in Dulbecco’s Modified Eagle Medium (DMEM, Gibco, USA) and supplemented with 10% fetal bovine serum (FBS, Gibco, Australia) at 37 °C in a 5% CO_2_ humidified incubator. Rabies virus CVS-11 strain (GenBank No. GQ918139.1) and street virus BD06 strain were propagated in BHK-21 cells and used throughout the experiments. Rabies virus SC16, GD1, NM3, QH2, LY, YN3, and XZ17 strains belonged to China I–VII clades, respectively. The background information of representative strains are shown in Table 1.

**Table 1:** Rabies virus background information

NO.	GenBank	Province	Host	Date of isolation	Lineage	Percent identity
SC16	AFM52619 (CSC1016D)	Sichuan	Dog	2010	China I	94.66
GD1	ADD39216 (GDZQ45)	Guangdong	Dog	2007	China II	97.14
NM3	AGV80474 (CNM1103C)	Inner Mongolia	Cow	2011	China III	94.08
QH2	AKA65932 (CQH1202D)	Qinghai	Dog	2012	China IV	93.51
LY	AAG34722 (CNX8601)	Ningxia	Human	1986	China V	95.42
YN3	AGK23495 (CYN1009D)	Yunnan	Dog	2010	China VI	94.85
XZ17	AYE21285 (CXZ1704H)	Tibet	Human	2018	China VII	94.08

SC16, GD1, NM3, QH2, LY, YN3, and XZ17 are the representative strains of clades China I–VII, respectively.

Percent Identity is compared with the glycoprotein (RABV-G) of the CTN-1 strain (GenBank No. ACR39382.1).

### Vaccine

The development and characterization of mRNA vaccine (LVRNA001) were previously described^23^. Briefly, the mRNA encoding the rabies virus glycoprotein (RABV-G) of the CTN-1 strain (GenBank No. ACR39382.1) was produced by in vitro transcription (IVT) and encapsulated using lipid nanoparticle (LNP) technology. Inactivated rabies virus vaccines were purchased from Liaoning Chengda Biotechnology Co., Ltd. (Lot number 4150466A human use, freeze-dried, 1 dose ≥2.5 IU).

### Animal ethics statement

This study was carried out in strict adherence to recommendations described in the Guide for the Care and Use of Laboratory Animals, the Office of Animal Welfare, and the United States Department of Agriculture. The procedures used for anesthesia and euthanasia of study animals followed tenets of the ARRIVE reporting guidelines^39^. Dogs and cynomolgus macaques were anesthetized with intramuscular injection of ketamine (10 mg/kg, 50 mg/mL) followed by intramuscular injection of xylazine hydrochloride (2 mg/kg, 20 mg/mL). Then, the animals were euthanized through exsanguination via the femoral artery. Mice were euthanized using carbon dioxide inhalation. For Sprague-Dawley rats, F0-generation male rats were anesthetized with isoflurane inhalation and then euthanized through exsanguination via the abdominal aorta and/or posterior vena cava. F0-generation female rats and F1-generation rats were euthanized using carbon dioxide inhalation. All procedures were carried out under trained personnel and under the supervision of veterinary staff.

### Pre-exposure vaccination against RABV infection in dogs

Dogs (4–6 month-old beagles) were obtained from the Institute of Military Veterinary Medicine, Academy of Military Medical Sciences (Changchun, China). All experiments were approved by the Research Ethics Committee of the College of the Institute of Military Veterinary Medicine, Academy of Military Medical Sciences (IACUC of IAC22W002). Dogs were fed and watered regularly twice a day under normal conditions before being challenged with the virus. The challenged dogs were observed and raised in the designated animal safety laboratory. Dogs were randomized by gender, age, and weight into four experimental groups, and the study was performed in a double-blind randomized fashion. Six dogs per group were vaccinated i.m. with LVRNA001 (10 or 50 μg, 0d/7d), inactivated vaccine (human use, 1 dose ≥2.5 IU, 0d/7d/21d) in 500 μL, or phosphate-buffered saline (PBS) (500 μL, 0d/7d). Dogs were challenged with 50-fold LD_50_ of street virus BD06 strain in the skeletal muscle of opposite hind legs on D35 (35 days post vaccination). Blood samples were collected on D7, D9, D11, D35, and D125 for neutralization antibody tests. Observation of animal survival continued through 125 days after vaccination. The experimental endpoint of this study was 125 days after the first vaccination.

### Post-exposure vaccination against RABV infection in dogs

Dogs (4–6-month-old beagles) were injected i.m. with 50-fold LD_50_ of virulent RABV-BD06 strain in the biceps femoris of the hindlimb. Six hours after infection, dogs were i.m. vaccinated with LVRNA001 (10 or 50 μg, 0d/7d), inactivated vaccine (human use, 1 dose ≥2.5 IU, 0d/3d/7d/14d/28d) in 500 μL, or PBS (500 μL, 0d/7d). Animal survival was evaluated until 3 months after the first dose. Blood samples were collected on D90 and used for neutralization antibody tests. The experimental endpoint of this study was 90 days after the first vaccination.

### Pre-exposure and post-exposure vaccination against different rabies strains in mice

Mice (BALB/c, female, 6 weeks old) were obtained from Huafukang Biotechnology Co., Ltd. (Beijing). The IACUC approval number was 2023-CCDC-IACUC-009. The animals were housed under specific pathogen-free conditions and a standard light cycle (12 h light/dark cycle).

In the pre-exposure prophylaxis murine model, ten mice per group were vaccinated i.m. with LVRNA001 (5 μg/high-dose group, 0d or 0d/7d), LVRNA001 (1.67 μg/low-dose group, 0d or 0d/7d), or inactivated vaccine (1/10 or 1/30 human use, 0d/7d). On Day 14 after the first vaccination, mice were challenged with 50-fold LD_50_ of RABVs (SC16, GD1, NM3, QH2, LY, YN3, and XZ17, belonging to China I–VII clades, respectively) delivered i.m. Observation of animal survival continued through 42 days after immunization. The experimental endpoint of this study was 42 days after the first vaccination.

In the post-exposure prophylaxis murine model, mice were injected i.m. with 50-fold LD_50_ of RABVs (SC16, GD1, NM3, QH2, LY, YN3, and XZ17, belonging to China I–VII clades, respectively). Two hours after infection, ten mice per group were vaccinated with LVRNA001 (5 μg/high-dose group, 0d/7d or 0d/14d), LVRNA001 (1.67 μg/low-dose group, 0d/7d or 0d/14d) or inactivated vaccine (1/10 or 1/30 human use, 0d/3d/7d/14d/28d). Mice were monitored for 35 days. Animal survival was evaluated 28 days after immunization. The experimental endpoint of this study was 35 days after the first vaccination.

### Immune generation period and immunodynamic testing in mice

Groups of six mice (BALB/c, female, 6 weeks old) were vaccinated i.m. with LVRNA001 (5 μg, 0d or 0d/7d or 0d/14d) or inactivated vaccine (1/10 human use, 0d/3d/7d/14d/28d). Blood samples were collected on D3, D5, D7, D10, D14, D21, D28, and D42 after the first vaccination. Neutralizing antibody titers were detected in sera by rapid fluorescent focus inhibition test (RFFIT). The experimental endpoint of this study was 42 days after immunization.

### Rapid fluorescent focus inhibition test (RFFIT)

Sera was inactivated by heating at 56 °C for 30 min, then serially diluted in a 96-well plate with human rabies immunoglobulin standards. CVS-11, at a dose that caused 80% infection of BSR cells in each well, was incubated with the serum for 1 h at 37 °C. Then BSR cells were added to each well and incubated for 24 h at 37 °C in 5% CO_2_. Finally, the cells were fixed and stained with a fluorescein isothiocyanate-conjugated antibody (Fujirebio, Cat.800-092) at a 1:50 dilution. The percentage of infected cells at each serum dilution was detected in each well using a fluorescence microscope. The RVNA titer (50% neutralization, ED50) of a test sample was mathematically interpolated using the Reed and Muench method^40^ and was calibrated and converted into IU/mL against the WHO SRIG (standard rabies immune globulins). Sera with a neutralizing antibody titer ≥0.5 IU/mL were considered positive.

### Single-dose acute toxicity study in CD-1 mice

A total of 30 CD-1 mice (half male and half female) were randomly divided into 3 groups (*n* = 10, half male and half female): negative (Saline), LVRNA001 (10 μg, low-dose group), and LVRNA001 (50 μg, high-dose group), which were injected into the thigh of the right hindlimb on D0. Then, the mice were continuously monitored for 14 days. Body weight was measured and weight gain rate was calculated on D-1 (1 day before administration), D3, D5, D7, D9, D11, and D14. The IACUC approval number was S-ACU22-0994. The experimental endpoint of this study was 14 days after immunization.

### Vaccination of cynomolgus macaques

According to the ICH Guideline S6 (R1) issued in 2011, cynomolgus monkey is recognized as an appropriate animal model for toxicological studies of biological products that have been or are intended to be used in humans. A total of 40 cynomolgus macaques aged 2.8 to 4.4 years, weighing from 2.16 to 4.48 kg, half male and half female, were purchased from Xiongsen Primate Laboratory Animal Breeding Development Co. Ltd. (Guangxi, China). Animals were included in the study after quarantine (Laboratory Animal Production License No: SCXK (Gui) 2021-0004, Laboratory Animal Quality certificate No: 0002990). The IACUC approval number was S-ACU22-1084. The animals were housed in stainless steel cages, with males and females separated into different cages. Each cage did not exceed five animals per gender per group. The temperature in the animal room was controlled within the range of 18–26 °C, with a relative humidity maintained between 40 and 70%. The vivarium light cycle was set at 12:12 h of light:dark. All animals were fed a non-human primate diet, which is nutritionally complete. The diet was supplemented with a variety of fruits and vegetables at a minimum of three times each week.

Cynomolgus macaques were randomly divided into 4 groups (*n* = 10, half male and half female): Saline group (negative control), Empty LNP group, LVRNA001 (50 μg) (low-dose group), and LVRNA001 (150 μg) (high-dose group), which were injected into the thigh of the right hindlimb on D0 (0-day post vaccination), D7, D21, and D35. The administration volume of the test product was 0.5 mL per animal. Cynomolgus macaques experienced repeated administration for 5 weeks, followed by a 4-week convalescence period. The cynomolgus macaques were observed for a total of 9 weeks. Cynomolgus macaques were clinically evaluated throughout the duration of the experiment, and body weight and temperature were monitored. The first six animals in each group were euthanized and used for histopathological examination on D39 (4 days after the administration of the last dose), whereas the remaining animals continued to be monitored during the convalescence period. Blood samples were collected on D-3 (3 days before administration), 4 h, 24 h, D4, D7, D11, D21, D25, D35, D37, D39, and D64, and used to measure neutralization antibodies, lymphocyte subsets, and biochemical indexes. The experimental endpoint of this study was 64 days after the first vaccination.

### Fluorescent antibody virus neutralization test (FAVN)

The titers of rabies virus-neutralizing antibodies (RVNAs) were determined using FAVN assays according to standards GB/T 34739-2017. Briefly, 3-fold dilutions of heat-inactivated sera were incubated with 50 µl suspension containing 50% tissue culture infectious dose (TCID_50_) of rabies virus CVS-11 strain for 1 h at 37 °C in 96-well plates. Then, 50 µl BHK-21 cells (4 × 10^5^/mL) were added to each well and cultured in a 5% CO_2_ incubator at 37 °C for 48 h. The cells were fixed with 80% ice-cold acetone, and stained with a RABV-specific monoclonal antibody (homemade) at a 1:200 dilution followed by FITC-conjugated goat anti-mouse IgG. Fluorescence in each well was detected with a fluorescence microscope, and the titers of RVNAs were calculated as IU/mL in order to be compared against the WHO standard (0.5 IU/mL).

### Biochemical evaluation of cynomolgus macaques

Venous blood from the hind limbs of cynomolgus macaques was collected into a heparinized tube. Plasma was separated at 1500 g and assayed to quantify the levels of fibrinogen (FIB) using a coagulation analyser (CS-5100, Sysmex Europe), aspartate aminotransferase (AST), alanine aminotransferase (ALT), alkaline phosphatase (ALP), albumin/globulin ratio (A/G), and C-reactive protein (CRP) using a Clinical Chemistry Analyzer (TBA-120FR, Canon).

### Analysis of lymphocyte subsets

To investigate lymphocyte subsets in cynomolgus macaques, blood was collected for peripheral blood mononuclear cells (PBMC) preparation. The PBMCs were stained with 10 µl of different combinations of PerCP mouse anti-human CD3 (BD Biosciences, Cat. 552851, 1/5 final dilution), FITC anti-human CD4 (Biolegend, Cat. 317408, 1/20 final dilution), APC anti-human CD8a (Biolegend, Cat. 301049, 1/20 final dilution), and PE anti-human CD20 (Biolegend, Cat. 302306, 1/20 final dilution) antibodies and incubated for 15 min at room temperature. Then, cells were detected on BD FACSCantoTM^II^ (BD Bioscience) and data were analyzed using FlowJo software (Tree Star).

### IFN-γ enzyme-linked immunospot (ELISpot) assays

PBMCs collected from the immunized cynomolgus macaques on D39 were prepared in 96-well ELISpot plates. The PBMCs were stimulated with RABV-G protein peptide pools (101 overlapping 15-mer peptides with 11-amino acids overlap, 2 µg/peptide/mL) for 21 h at 37 °C. IFN-γ secreting cells were visualized using an ELISpot assay kit (Cat. 3421M-4APW-10, MabTech, Sweden) according to the manufacturer’s instructions. The resulting spot-forming cells (SFCs) were counted with an ELISpot reader (ImmunoSpot S6, CTL).

### Histopathological examination

For histopathological examination, 54 tissues were collected, including brain, heart, liver, spleen, lung, kidney, injected muscle, eyeball, optic nerve, and testis. The eyeball, optic nerve, and testis were fixed in Davidson’s fixative, and all other tissues were fixed in a 10% neutral formalin solution. Tissues were embedded in paraffin, sectioned, and stained with hematoxylin and eosin (HE). The sections were examined and photographed by a pathologist. The data were analyzed by the Provantis system (SAS 9.4 statistical software with mean ± standard deviation).

### Reproductive toxicity study in Sprague-Dawley rats

Sprague-Dawley rats were group housed (up to 4 per cage) in single-sex groups until paired for mating, at which time females were housed 1:1 with a nontreated breeding male. The female rats were individually housed through gestation and lactation following evidence of mating. Rats were provided with a complete rodent breeding diet and locally sourced water. Environmental conditions throughout the studies were set to maintain a relative humidity of 40–60% and temperature of 22–25 °C, along with the room lighting set to provide a 12 h light/dark cycle. All animal care and experimental procedures were conducted in compliance with guidelines for the care and use of laboratory animals and the relevant regulations of the Institutional Animal Care and Use Committee (IACUC) and approved by the IACUC (approval number: S-ACU22-1431).

A total of 336 Sprague-Dawley rats (224/female, 112/male) were randomly divided into four groups according to body weight, with 28 males and 56 females in each group. The four groups were the Saline group (negative control, 0.5 mL/ rat), empty LNP group (0.5 mL/ rat), LVRNA001 (25 μg) (low-dose group, 0.25 mL/ rat), and LVRNA001 (50 μg) (high-dose group, 0.5 mL/ rat), which were injected into the left hind limb muscle. The study was split into two phases: an EFD phase and a littering phase. EFD phase F0 females (*n* = 28/group; the number of pregnant rats in groups 1–4 were 28, 28, 27, and 25, respectively) were vaccinated i.m. with LVRNA001 (25 or 50 μg), Empty LNP, or PBS on D14 (28 days prior to pairing for mating), D21, D35, and GD6. Rats were terminally anesthetized and dissected for fetal examination on GD20. After mating, littering phase F0 females (*n* = 28/group; the number of pregnant rats of groups 1–4 were 27, 25, 25, and 21, respectively) were additionally dosed on postnatal day PND7. Rats were used for normal delivery until the end of PND21. F0 males (*n* = 28/group) were administrated on D0, D7, D21, and D35. Mating behavior was facilitated on D42. In the EFD phase, the number of pregnancies, uterine and placenta weight, number of live fetuses, and rate of dead fetuses were monitored. In the littering phase, the number of pregnancies, total pups on PND0, the number of pups that survived on PND0, live pups retained on PND4, birth index (the proportion of pups who were born alive), and lactation index (the proportion of pups alive on PND4 who were alive on PND21) were monitored and recorded. The IACUC approval number was S-ACU22-1431.

### Statistical analysis

The data were statistically analyzed by GraphPad Prism (8.0) and presented as the means ± standard deviation. Comparisons among groups were performed using an ordinary one-way or two-way analysis of variance (ANOVA) test followed by Tukey’s test. A *p* value less than 0.05 was considered significant.

### Supplementary information

Supplementary Information file

## Data Availability

All data upon which conclusions were drawn are included in the publication article.
