# Supplementary material for: Safety and efficacy assessment of an mRNA rabies vaccine in dogs, rodents, and cynomolgus macaques
Source: NPJ Vaccines. 2024 Jul 20;9:130. doi: 10.1038/s41541-024-00925-w (PMC11271276; doi:10.1038/s41541-024-00925-w)
Supplement: Supplementary file 1 — Supplementary Information file [file 41541_2024_925_MOESM1_ESM.pdf]

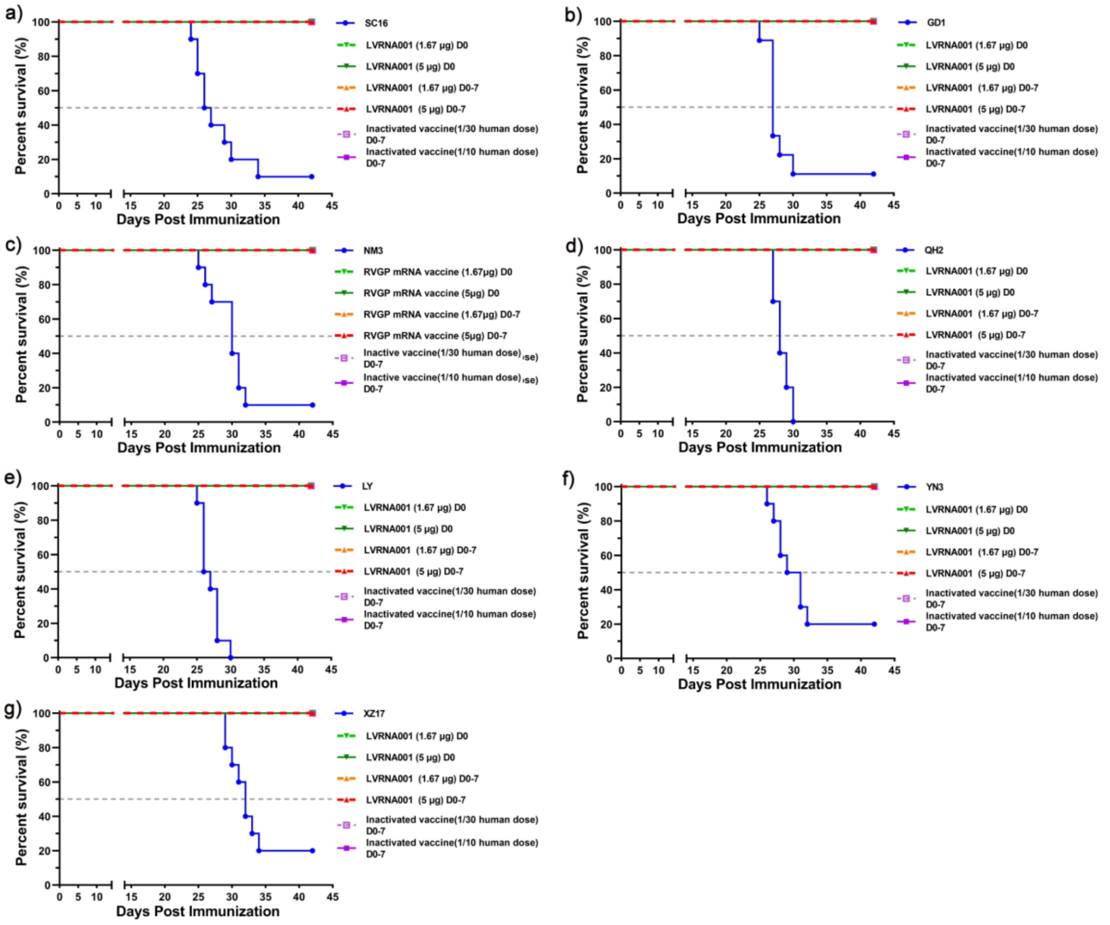

Supplementary Fig.1 The animals were monitored daily and survival rates were calculated in a pre-exposure mouse model

Mice were vaccinated i.m. with LVRNA001 (5 µg, 0d or 0d/7d), LVRNA001 (1.67 µg, 0d or 0d/7d), or inactivated vaccine (1/10 or 1/30 human use, 0d/7d), then challenged i.m. with 50-fold LD_{50} of RABVs (SC16, GD1, NM3, QH2, LY, YN3, and XZ17) on day 14. **a-g** The animal survival was monitored daily for 42 days after challenge, survival rates of different strains were calculated (n=10).

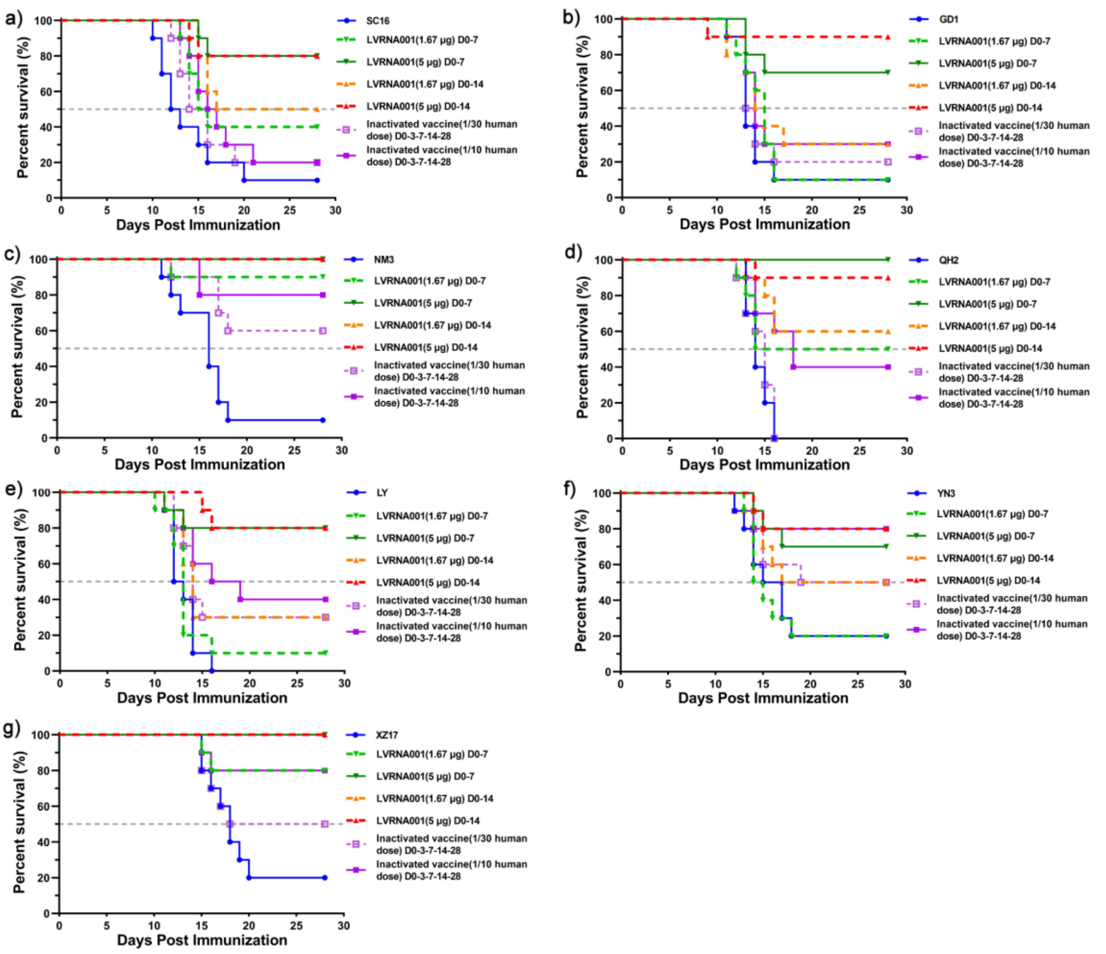

Supplementary Fig.2 The animals were monitored daily and survival rates were calculated in a post-exposure mouse model

Mice were first injected i.m. with 50-fold LD_{50} of RABVs (SC16, GD1, NM3, QH2, LY, YN3, and XZ17), two hours later, they were immunized i.m.with LVRNA001 (5 µg, 0d/7d or 0d/14d), LVRNA001 (1.67 µg, 0d/7d or 0d/14d) or inactivated vaccine (1/10 or 1/30 human use, 0d/3d/7d/14d/28d). **a-g** The animal survival was monitored daily for 28 days after viral challenge, survival rates of different strains were calculated (n=10).

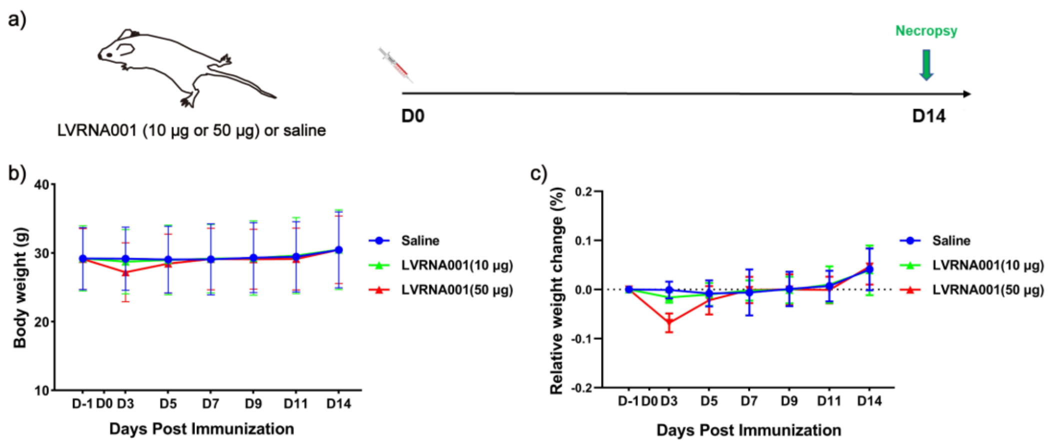

Supplementary Fig.3 LVRNA001 had no adverse effects in an acute toxicity study in mice

a Schematic diagram depicting an acute toxicity study schedule for mice. Mice were immunized with a low dose (10 μg) or high dose (50 μg) of LVRNA001, or PBS on D0. Mice were continuously observed through 14 days post immunization. **b** Body weight was measured on D-1 (1 days before administration), D3, D5, D7, D9, D11, and D14. **c** Weight gain rate was calculated 14 days post immunization. n=10. All error bars represent standard deviation. Statistical analysis was performed using an ordinary one-way ANOVA.

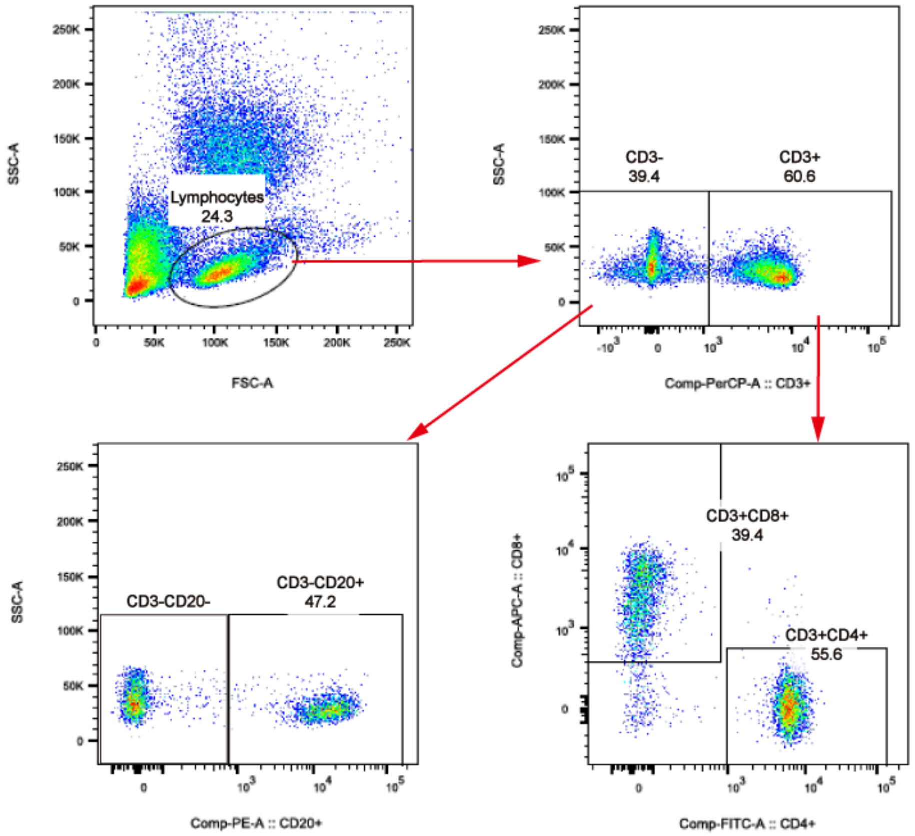

Supplementary Fig.4 The gating strategy for the flow cytometry
